# Supplementary material for: Modulation of Inflammasome and Pyroptosis by Olaparib, a PARP-1 Inhibitor, in the R6/2 Mouse Model of Huntington’s Disease
Source: Cells. 2020 Oct 13;9(10):2286. doi: 10.3390/cells9102286 (PMC7602058; doi:10.3390/cells9102286)
Supplement: Supplementary file 1 [file cells-09-02286-s001.pdf]

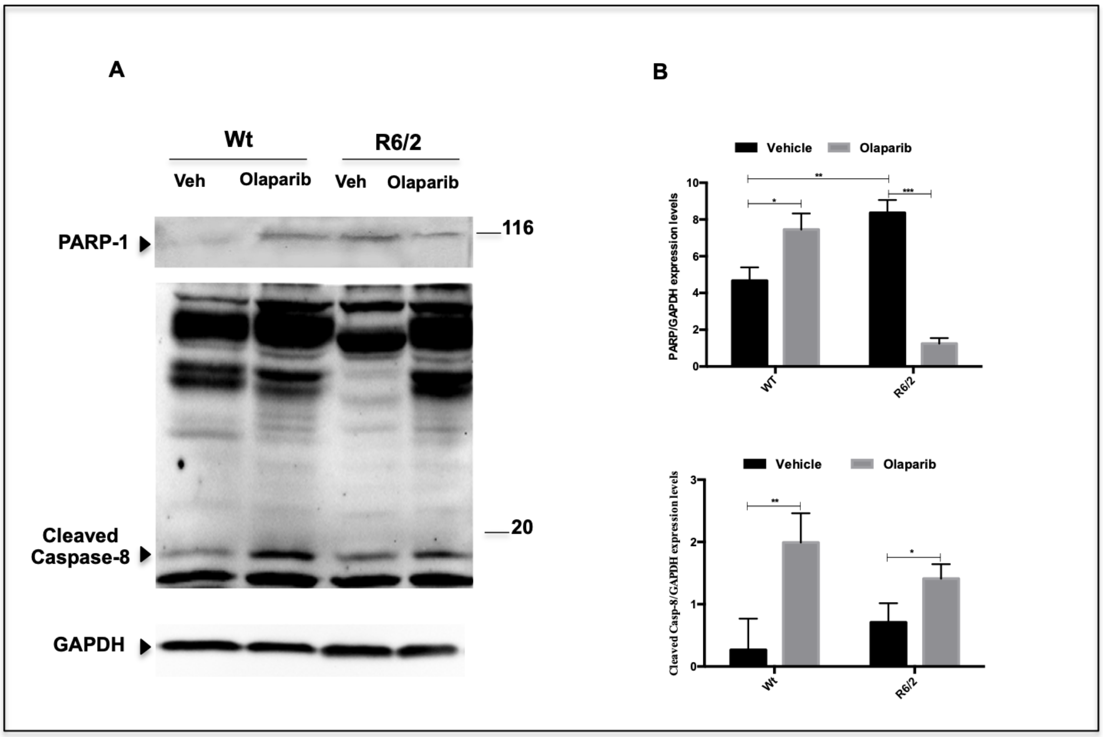

Figure S1. Effects of Olaparib on full-length PARP expression levels and caspase-8 activation (**A**) Brain tissue homogenates of all experimental groups were subjected to Western blotting using an antibody that specifically recognizes the cleaved product of caspase-8. (**B**) Sidak multiple comparison tests showed a significant down-modulation of PARP expression levels in the Olaparib-treated R6/2 mice. Moreover, Western blot data revealed a significant up-regulation of PARP in the Olaparib-treated Wt mice, amenable to a condition of chronic oxidative stress that the drug could cause in an unperturbed organism. Moreover, Two Way ANOVA statistical analysis revealed that 13-week-old vehicle treated Wt and R6/2 mice had very low levels of cleaved caspase-8 compared to the Olaparib-treated mice group, in which, as previously described, the drug promoted a moderate increase of death receptors and caspase-8 activation ($p < 0.01$ and $p < 0.05$, respectively).
